# Information Theory Applications in Signal Processing

**DOI:** 10.3390/e21070653

**Published:** 2019-07-03

**Authors:** Sergio Cruces, Rubén Martín-Clemente, Wojciech Samek

**Affiliations:** 1 Departamento de Teoría de la Señal y Comunicaciones, Universidad de Sevilla, Camino de los Descubrimientos s/n, 41092 Seville, Spain; 2 Fraunhofer Heinrich Hertz Institute HHI, 10587 Berlin, Germany

**Keywords:** information theory applications, signal processing, communications, machine learning, data analysis, image and audio processing

The birth of Information Theory, right after the pioneering work of Claude Shannon and his celebrated publication of the paper “A mathematical theory of Communication” [1], was a milestone that fuelled the posterior development of modern communications. Since its origins, this discipline has progressively expanded its influence on statistical signal processing, a closely related research field.

Information Theory has contributed to the statistical foundations and clarification of key concepts or underlying limits, not only in communications [2,3], but also in several other signal processing areas, such as: time series analysis [4], estimation theory [5,6], detection theory [7], machine learning [8], statistical modeling [9], image and multimedia processing [10], speech and audio processing [11,12], neural networks [13] and deep learning systems [14,15]. All of these are dynamic and fast-growing research fields that have to cope with increasingly complex scenarios and novel applications. Hence, as it was stated in the invitation to the Special Issue: “… there is a need for specific information theoretic criteria and algorithms that work in each of the considered situations and attain a set of desired goals, for instance, an enhancement in the interpretability of the solutions, improvements in performance, robustness with respect to the model uncertainties and possible data perturbations, a reliable convergence for the algorithms and any other kind of theoretical guarantees”.

Given the previously described context, the main focus of this Special Issue is on recent developments in information theory applications for advanced methods in signal processing. The selected collection of works comprises seventeen original contributions that span a wide range of signal processing topics. We briefly summarize below their scope and contents.

Five of the articles [16,17,18,19,20] are devoted to the analysis of the latent components of the observations. In [16], the authors suggest a nonlinear estimation of the partial correlation coefficients, due to its potential applications in graph signal processing. These estimates, which assume an underlying non-Gaussian density model based on a mixture of mutually independent components, better capture the connectivity of the graph in this kind of scenarios.

The works in [17,18] present some unsupervised learning criteria based on the family of Alpha–Beta divergences and validate them through both synthetic and real experiments. These generalized dissimilarity measures are governed by the two real parameters, α and β, that smoothly connect several classical divergences (see [21]). In [17], Sarmiento et al. obtain the closed-form expressions for the αβ-centroids, and propose an original αβ-*k*-means algorithm for centroid-based clustering. Delmaire et al. propose in [18] several informed and weighted αβ-non-negative matrix factorization methods. The contribution reveals that the combination of the factorization criterion with a set of structural constraints (which are derived from some available information in the chemical source apportionment problem) enhances the robustness of the decomposition with respect to possible contaminations by noise and outliers.

In [19], Pinchas studies the problem of finding closed-form approximated expressions for the conditional expectation of blind adaptive deconvolution problem. She proposes an estimate that improves the one obtained via the maximum entropy density approximation technique and that continues to be reliable for low signal-to-noise ratios, whereas, in [20], Wu et al. address the application of an enhanced combination of compressed sensing with information based techniques for tracking the health monitoring of a machinery process and the prediction of its remaining useful life.

Within machine learning, ensemble learning techniques exploit the integration of multiple algorithms to obtain a better or more robust predictive performance. In [22], Vigneron et al. analyze the optimal combination rule for an ensemble of weak rank classifiers. Their aim is to obtain an improved decision and also to propose a suitable information measure for quantifying their degree of consensus.

Data and image analysis are also important areas of research in which information theory has found practical applications on an everyday basis. In the context of image and video processing, Szczęsna [23] puts forward a new form of approximate entropy, specifically designed for quaternion time series, as well as its application to the analysis of human gait kinematic data, where quaternions are used to represent rotations. Zhou et al. [24] present a dictionary learning algorithm, based on brightness and detail clustering, as inspired by the characteristics of the human visual system, for medical image fusion. In [25], Ballesteros et al. propose variable length codes, based on Collatz conjecture, for transforming images into non-intelligible audio, aiming in this way at providing privacy to image transmissions through an encryption scheme. Finally, in the field of acoustic signal processing, Shen et al. [26] develop an auditory inspired convolutional neural network that simulates the processing procedure of the human auditory system in discriminating ships of different classes from raw hydrophone data.

The remaining articles of this special issue [27,28,29,30,31,32,33] have topics within area of communications. On the one hand, the characterization of the achievable rate region of a given dual-hop multiple-access relay network, under linear beamforming, is analyzed in [27]. On the other hand, the authors of [28] study the sum-rate of multi-user MIMO systems for correlated Rayleigh fading channels, in the specific situation of multi-cell pilot contamination. Among the results of this contribution, we can mention the lower bound of the sum-rate, an approximation of its outage probability, and also of its asymptotic performance for a sufficiently large number of antennas at the base station.

We now turn to the field of coding theory: Zhang et al. [29] develop several novel types of maximum-distance separable self-dual codes over finite fields with odd characteristics, being based on a generalization of Reed–Solomon codes. In [30], Wang et al. analyze the Turbo Decoding Message Passing (TDMP) algorithm from the perspective of density evolution and Gaussian approximation, thus providing new theoretical foundations for this method. In addition, based on a certain normalized factor, the authors propose a simplified TDMP algorithm with improved performance.

Another exciting area of research covered in this special issue deals with radar signal processing. Two papers address the problem of designing the waveforms transmitted by the radar: in [31], Wang et al. propose a technique to maximize the signal-to-interference-plus-noise ratio (SINR) for known- and random-target models, and the mutual information (MI) between the radar echos and the random-target spectrum responses. In [32], Hao et al. present a cognitive waveform design method subject to a peak-to-average power ratio constraint. It is noteworthy that these authors develop a minorization–maximization technique to reduce the computation burden of the overall procedure.

Finally, bringing this brief outline to an end, we would also like to mention that block ciphering, an important subarea of cryptography, has been addressed in [33] by Wang et al., which present a novel algorithm that combines chaotic theory and Feistel block ciphers for securing the transmission of texts. 
